# Individual-, household-, and community-level factors associated with eight or more antenatal care contacts in Nigeria: Evidence from Demographic and Health Survey

**DOI:** 10.1371/journal.pone.0239855

**Published:** 2020-09-25

**Authors:** Michael Ekholuenetale, Faith Owunari Benebo, Ashibudike Francis Idebolo

**Affiliations:** 1 Department of Epidemiology and Medical Statistics, Faculty of Public Health, College of Medicine, University of Ibadan, Ibadan, Nigeria; 2 Clinical Case Management Unit, Management Sciences for Health, Abuja, Nigeria; 3 Department of International Public Health, Liverpool School of Tropical Medicine, Pembroke Place, Liverpool, United Kingdom; University of Botswana, BOTSWANA

## Abstract

**Introduction:**

Antenatal care (ANC) is a vital mechanism for women to obtain close attention during pregnancy and prevent death-related issues. Moreover, it improves the involvement of women in the continuum of health care and to survive high-risk pregnancies. This study was conducted to determine the prevalence of and identify the associated factors of eight or more ANC contacts in Nigeria.

**Methods:**

We used a nationally representative cross-sectional data from Nigeria Demographic and Health Survey—2018. A total sample of 7,936 women were included in this study. Prevalence was measured in percentages and the factors for eight or more ANC contacts were examined using multilevel multivariable binary logistic regression model. The level of significance was set at P<0.05.

**Results:**

The prevalence of eight or more ANC contacts in Nigeria was approximately 17.4% (95% CI: 16.1%-18.7%). Women with at least secondary education were 2.46 times as likely to have eight or more ANC contacts, when compared with women with no formal education. Women who use media were 2.37 times as likely to have eight or more ANC contacts, when compared with women who do not use media. For every unit increase in the time (month) of ANC initiation, there was 53% reduction in the odds of eight or more ANC contacts. Rural women had 60% reduction in the odds of eight or more ANC contacts, when compared with their urban counterparts. Women from North East and North West had 74% and 79% reduction respectively in the odds of eight or more ANC contacts, whereas women from South East, South South and South West were 2.68, 5.00 and 14.22 times respectively as likely to have eight or more ANC contacts when compared with women from North Central.

**Conclusion:**

The coverage of eight or more ANC contacts was low and can be influenced by individual-, household-, and community-level factors. There should be concerted efforts to improve maternal socioeconomic status, as well as create awareness among key population for optimal utilization of ANC.

## Introduction

The fifth Millennium Development Goal (MDG-5) sought to reduce maternal mortality by 75% between 1990 and 2015 [1]. However, while there were significant improvements such as a 45% reduction in maternal mortality ratio, and a 12% increase in skilled birth attendance by 2015, death from pregnancy and childbirth continued globally [2]. Transitioning from the MDGs in 2015 [3], the Sustainable Development Goals (SDGs) aim to reduce maternal mortality by 2030 to less than 70 per 100,000 live births [4]. However, in 2015, about 303,000 deaths occurred around the world resulting from pregnancy and childbirth, with a majority of these deaths occurring in low resource settings [2,5]. This staggering estimate in maternal mortality contravenes the third goal in SDGs which focus to ensure healthy lives and promote well-being for all at all ages [4].

Sub-Saharan Africa (SSA) disproportionately contributes to global maternal death, where about 196,000 maternal deaths occurred in 2015 [2]. The lifetime risk of dying from pregnancy and childbirth is 1 in 37 in resource-constrained settings as compared with high-income countries, where the lifetime risk of maternal death is 1 in 7,800 [2,5]. These figures raise important, yet critical questions as to whether any progress has been achieved in SSA thus far. Interestingly, ANC remains a prominent measure for improving maternal health outcomes and provided by skilled births attendants to ensure positive health outcomes for mothers and children [6]. In 2002, WHO recommended a goal-orientated approach to ANC to improve quality of care and increase ANC coverage, specifically in resource-constrained settings [7]. The FANC model was commonly referred to as the basic ANC model and included four ANC visits occurring between 8 and 12 weeks of gestation, between 24 and 26 weeks, at 32 weeks, and between 36 and 38 weeks respectively [7]. Recognizing the benefits of increased ANC contacts, in 2016, the World Health Organization (WHO) recommended eight ANC contacts in the place of focused ANC (FANC) model, to ensure better pregnancy experience [8].

Although Nigeria adopted the previous WHO FANC model, which recommended one visit during each trimester of gestation and a final visit immediately preceding delivery for women without pregnancy-related complications or risk factors, still only 52% of women made all four recommended visits in 2013 [9]. This raises concerns regarding the adoption and implementation of the WHO new guideline, which recommends that the number of contacts for a woman be increased from four to eight.

In spite of the FANC model institutionalized in-country over a decade, it is worrisome to note that Nigeria currently accounts for about 20% of global maternal deaths [10]. Moreover, numerous factors have been attributed to the underutilization of ANC services, such as far distance of the health facilities from clients, lack of transportation and out-of-pocket spending for healthcare, maternal age, religious background, parity, health insurance coverage, ethnicity, marital status, involvement in labour force, education and accessibility to health information [11–16]. In addition, multifactorial inequalities in access to healthcare, impact of environmental, economic, socio-demographic and political factors have contributed to poor health outcomes for women of reproductive age especially in poor resource settings [17]. Studies have identified community-level factors associated with ANC visits, these include rural-urban residential status, years of education/rate of women’s literacy, income score/ poverty rate, geographical region, community exposure to newspaper, television, community education and wealth concentration, antenatal care service availability and readiness [13,18,19]. Largely, these factors were examined to determine their association with eight or more ANC contacts in Nigeria. To the best of our knowledge, there is no study on the coverage and factors of eight ANC in Nigeria that used data collected after the launched of the WHO new guideline. In this study, we aimed to assess the individual, household and community-level factors associated with eight or more ANC contacts.

## Methods

### Data source

We used a nationally representative cross-sectional data. The individual woman questionnaire in Nigeria Demographic and Health Survey (NDHS) was analyzed in this study. A total sample of 7,936 women of reproductive age who became pregnant and had given birth after the new guideline of eight ANC contacts was endorsed were included in this study. The 2018 NDHS is the sixth survey of its kind to be implemented by the National Population Commission (NPC). Data collection took place from 14 August to 29 December 2018. The sample was selected using a stratified, two-stage cluster design, with Enumeration Areas (EAs) as the sampling units for the first stage. The complete listing of households carried out in each of the 1,389 selected EAs, an approximate number of 30 households was selected in every cluster resulting to a total of 41,821 women were interviewed during the survey, yielding a response rate of 99%. A total sample of 7,936 women of reproductive age who became pregnant and had given birth after the new guideline of eight or more ANC contacts was endorsed by WHO [8], were included in this study.

In particular, NDHS 2018 used a three-stage sampling stratification, in which respondents were first stratified by urban versus rural dwelling, and EAs were then selected randomly within each stratum. Finally, households within each EA were then selected for the survey using equal probability sampling. This three-stage sampling method was taken into account in the computation of survey weights, applied to ensure the representativeness of the sample with regard to the general population. The sampling frame used for the 2018 NDHS is the Population and Housing Census of the Federal Republic of Nigeria (NPHC), which was conducted in 2006 by the National Population Commission. The sample for the 2018 NDHS was a stratified sample selected in two stages. Stratification was achieved by separating each of the 36 states and the Federal Capital Territory into urban and rural areas. In total, 74 sampling strata were identified. Data for this study are derived from the individual female data for analysis. The DHS project, funded primarily by the United States Agency for International Development (USAID) with support from other donors and host countries, has conducted over 230 nationally representative and internationally comparable household surveys in more than 80 countries since its inception in 1984. The data is available in the public domain and accessed at; http://dhsprogram.com/data/available-datasets.cfm. Details of DHS sampling procedure has been reported previously [20].

### Variables selection and measurement

#### Outcome

The frequency of ANC contacts with doctors, nurses and midwives was measured dichotomously; less than eight ANC contacts vs. eight or more ANC contacts. The WHO ANC guideline recommendations mapped to the eight recommended contacts, presents a summary framework for the 2016 WHO ANC model in support of a positive pregnancy experience [8,21,22].

#### Individual-level factors

Family mobility: internal immigrant (if a respondent lived in the current location in less than 5 years) vs. native (if a respondent had lived in the current location at least 5 years). Religious background: Christianity, Islam and African Traditional Religion (ATR)/others. Literacy: cannot read at all, able to read only part of a sentence and able to read whole sentence. Total number of children ever born: 1–2, 3–4 and over 4 children. Women’s knowledge level was measured using; educational attainment, read newspaper/magazines, listen to radio, watch television and use internet [23]. Using Principal Component Analysis (PCA), the standardized z-score was used to disentangle the overall assigned scores to low, medium and high. Maternal educational attainment: no formal education, primary and secondary or higher education. Media use was measured dichotomously (yes vs. no) if a respondent used any or newspaper/magazine, radio, television or internet irrespective of the frequency levels, "almost every day", "at least once a week", and "less than once a week" as yes/use and the response level "not at all" as no/not use [24]. Maternal age: 15–19, 20–24, 25–29, 30–34, 35–39, 40–44, 45–49. Wanted child when became pregnant: then, later and wanted no more. Health insurance: covered vs. not covered. Marital status: never in union, currently married/living with a partner and formerly in union. Employment status: working vs. not working. Family type: monogyny vs. polygyny. Intimate partner violence: yes (if a woman had physical, sexual or emotional violence) vs. no (otherwise). Women’s autonomy was measured using PCA for selected items: person who usually decides on respondent's health care, person who usually decides on large household purchases and person who usually decides on visits to family or relatives [25]. The standardized z-score was then used to disentangle the overall assigned scores to low, medium and high. Time to ANC initiation (in months).

#### Household-level factors

Sex of household headship was male vs. female. Household size was based on the total number of individuals who resided together and grouped as: 1–4, 5–8 and over 8 persons. Household wealth quintiles: PCA was used to assign the wealth indicator weights. This procedure assigned scores and standardized the wealth indicator variables such as; bicycle, motorcycle/scooter, car/truck, main floor material, main wall material, main roof material, sanitation facilities, water source, radio, television, electricity, refrigerator, cooking fuel, furniture, number of persons per room. The factor coefficient scores (factor loadings) and z-scores were calculated. For each household, the indicator values were multiplied by the loadings and summed to produce the household’s wealth index value. The standardized z-score was used to disentangle the overall assigned scores to; poorest/poorer/middle/richer/richest categories [26,27]. In creating household wealth index, rural-urban differences was adjusted for and used in the analysis. As a response to criticism that a single wealth index is too urban in its construction and not able to distinguish the poorest of the poor from other poor households, the new variable created to provide an urban- and rural-specific wealth index was utilized.

#### Community-level factors

We used EAs to represent communities prominently because the DHS did not collect aggregate-level data at the community level. Hence, community-level variables included in the analysis were based on women’s characteristics particularly those that have implications for accessing ANC. Cultural norms about wife-beating was created by aggregating responses from women in each community. Here, we used the items: “beating justified if wife goes out without telling husband”, “beating justified if wife neglects the children”, “beating justified if wife argues with husband”, “beating justified if wife refuses to have sex with husband” and “beating justified if wife burns the food”. Finally, a binary variable was created for acceptance of wife beating [28]. Maternal residential status was measured as: urban vs. rural. Geographical region was categorized thus: North Central, North East, North West, South East, South South and South West.

Furthermore, aggregate community-level variables were constructed by aggregating individual level characteristics at the community (cluster) level and categorization of the aggregate variables was done as low or high based on the distribution of the proportion values calculated for each community. If the aggregate variable was normally distributed mean value and if not normally distributed median value was used as cut off point for the categorization. Community-level poverty was categorized as high if the proportion of women from the two lowest wealth quintiles in a given community was 43–100% and low if the proportion was 0–42%. Community-level media use was categorized as high if the proportion was 60–100% and as low if the proportion of women who use media in the community was 0–59%. Community-level illiteracy was categorized as high if proportion of women who cannot read at all was 67–100% and as low if the proportion of women who cannot read at all was 0–66%. Community-level urban residence was categorized as high if proportion of women who reside in urban area was greater than 1–100% and as low if the proportion of women who reside in urban area was 0%. Community-level women’s autonomy was categorised as high if the proportion of women who had at least moderate autonomy was 61–100% and categorized as low if the proportion was between 0–60%. This approach was used in a previous study [29,30].

### Ethical consideration

In this study, we utilized population-based secondary datasets available in public domain/ online with all identifier information removed. The authors were granted access to use the data by MEASURE DHS/ICF International. DHS Program is consistent with the standards for ensuring the protection of respondents’ privacy. ICF International ensures that the survey complies with the U.S. Department of Health and Human Services regulations for the respect of human subjects. No further approval was required for this study. More details about data and ethical standards are available at http://goo.gl/ny8T6X.

### Statistical analysis

The survey (‘svy’) module was used to adjust for stratification, clustering and sampling weights to compute the estimates of eight or more ANC contacts. The prevalence of eight or more ANC contacts was explored using percentage. A cut-off of 0.7 was used to determine multicollinearity known to cause major concerns in the logit model [31]. Consequently, maternal literacy and knowledge were excluded from the model as they were found to have positive interdependence with educational attainment which was therefore retained in the model. Other significant variables from Chi-square test or student’s t-test at 25% level of significance were retained in the logit model in the absence of multicollinearity.

A multivariable multilevel binary logistic regression model was used to estimate the fixed and random effects of the factors associated with eight or more ANC contacts. We specified a 3-level model for binary response reporting eight or more ANC contacts, for women (at level 1), in a household (at level 2) from an Enumeration Area (at level 3). We constructed five models. The first model, an empty or unconditional model without any explanatory variables, was specified to decompose the amount of variance that existed between community and household levels. The null or empty model is important for understanding the community and households’ variations, and we used it as the reference to estimate how much household and community factors were able to explain the observed variations. In addition, we used it to justify the use of multilevel statistical framework, because if the community variance was not significant in the empty model, it advised to use the single-level logistic regression. The second model contained only individual-level factors, the third model contained only household-level factors, and the fourth model contained only community-level factors. Finally, the fifth model simultaneously controlled for individual, household and community level factors (Full model). Statistical significance was determined at p< 0.05. The Bayesian and Akaike Information Criterions were used to select the best model out of the five models. A lower value on Akaike or Bayesian Information Criterion indicates a better fit of the model [32]. Data analysis was conducted using Stata Version 14 (StataCorp., College Station, TX, USA).

### Fixed and random effects

The results of fixed effects (measures of association) were reported as adjusted odds ratios (AORs) with their 95% confidence interval (CI). The probable contextual effects were measured by the Intra-class Correlation (ICC) and Median Odds Ratio (MOR) [33]. We measured the similarity between respondents in the same household and within the same community using ICC. The ICC represents the percentage of the total variance in the probability of eight or more ANC contacts that is related to the household and community level, i.e. measure of clustering of odds of eight or more ANC contacts in the same household and community. The MOR measures the second or third level (household or community) variance as odds ratio and estimates the probability of eight or more ANC contacts that can be attributed to household and community context. MOR equal to one indicates no household or community variance. Conversely, the higher the MOR, the more important are the contextual effects for understanding the probability of eight or more ANC contacts. The ICC was calculated by the linear threshold according to the formula used by Snijders and Bosker [34], whereas the MOR is a measure of unexplained cluster heterogeneity.

## Results

### Sample characteristics

Based on the results from Table 1, approximately 26.1% (95% CI: 23.1%-29.3%) of native women reported eight or more ANC contacts. Similarly, about 33.1% (95% CI: 30.7%-35.5%) of Christian women, 36.1% (95% CI: 33.5%-38.8%) of women who are able to read whole sentence, 23.0% (95% CI: 21.0%-25.1%) of women who had ever given birth to at most two children, 35.6% (95% CI: 33.1%-38.2%) of women with high knowledge, 34.0% (95% CI: 31.8%-36.3%) of women with at least secondary education, 25.2% (95% CI: 23.3%-27.0%) of women who use media had eight or more ANC contacts respectively. Furthermore, about 22.7% (95% CI: 20.0%-25.7%) of women aged 30–34 years, 39.3% (95% CI: 30.0%-49.4%) of women covered by health insurance, 21.1% (95% CI: 19.5%-22.9%) of women who participated in labour force, 21.0% (95% CI: 19.8%-23.0%) of women in monogyny, 24.0% (95% CI: 21.6%-26.7%) of women with no history of intimate partner’s violence, 31.3% (95% CI: 28.6%-34.1%) of women with high autonomy had eight or more ANC contacts respectively. See the table below for further details.

**Table 1:** Prevalence of eight or more ANC contacts across women’s characteristics, NDHS—2018.

Variable	Number of women (%)	Weighted eight or more ANC contacts; % (95%CI)	P
**Family mobility**			<0.001*
Internal immigrant	1267 (16.5)	26.1 (23.1–29.3)	
Native	6392 (83.5)	14.8 (13.5–16.2)	
**Religion**			<0.001*
Christianity	3048 (38.4)	33.1 (30.7–35.5)	
Islam	4820 (60.7)	9.1 (7.9–10.4)	
ATR/others	68 (0.9)	7.2 (1.5–27.8)	
**Literacy**			<0.001*
Cannot read at all	4678 (59.0)	7.2 (6.2–8.3)	
Able to read parts of sentence	1383 (17.4)	27.3 (24.2–30.7)	
Able to read whole sentence	1874 (23.6)	36.1 (33.5–38.8)	
**Number of children ever born**			<0.001*
1–2	2903 (36.6)	23.0 (21.0–25.1)	
3–4	2279 (28.7)	19.2 (17.1–21.6)	
4+	2754 (34.7)	10.2 (8.8–11.8)	
**Women’s knowledge**			<0.001*
Low	3059 (38.5)	2.8 (2.1–3.7)	
Moderate	2386 (30.1)	17.6 (15.5–19.8)	
High	2491 (31.4)	35.6 (33.1–38.2)	
**Educational attainment**			<0.001*
No formal education	3558 (44.8)	3.8 (3.0–4.8)	
Primary	1202 (15.2)	16.5 (13.8–19.5)	
Secondary+	3176 (40.0)	34.0 (31.8–36.3)	
**Media use**			<0.001*
No	3176 (40.0)	5.3 (4.3–6.4)	
Yes	4760 (60.0)	25.2 (23.3–27.0)	
**Women’s age**			<0.001*
15–19	698 (8.8)	8.0 (6.1–10.5)	
20–24	1871 (23.6)	14.9 (13.0–17.0)	
25–29	2258 (28.4)	18.7 (16.7–20.8)	
30–34	1598 (20.1)	22.7 (20.0–25.7)	
35–39	1004 (12.7)	18.7 (15.9–21.8)	
40–44	405 (5.1)	14.8 (10.7–20.0)	
45–49	102 (1.3)	11.5 (5.7–21.9)	
**Wanted child when became pregnant**			<0.001*
Then	6920 (87.2)	16.5 (15.2–18.0)	
Later	790 (10.0)	23.2 (20.1–26.7)	
No more	226 (2.8)	23.9 (17.4–32.0)	
**Health insurance**			<0.001*
Not covered	7789 (98.1)	17.0 (15.7–18.3)	
Covered	147 (1.9)	39.3 (30.0–49.4)	
**Marital Status**			0.847
Never in union	212 (2.7)	17.7 (12.4–24.8)	
Currently married/living with a partner	7588 (95.6)	17.3 (16.0–18.7)	
Formerly in union	136 (1.7)	19.6 (11.8–30.8)	
**Employment status**			<0.001*
Not working	2926 (36.9)	11.0 (9.6–12.6)	
Working	5010 (63.1)	21.1 (19.5–22.9)	
**Family type**			<0.001*
Monogyny	5345 (70.6)	21.0 (19.8–23.0)	
Polygyny	2222 (29.4)	8.2 (6.8–9.8)	
**Intimate partner violence**			0.066*
No	1471 (60.0)	24.0 (21.6–26.7)	
Yes	980 (40.0)	20.2 (17.4–23.4)	
**Women’s autonomy**			<0.001*
Low	3255 (42.9)	8.9 (7.6–10.2)	
Moderate	2156 (28.4)	16.3 (14.4–18.6)	
High	2177 (28.7)	31.3 (28.6–34.1)	
**Time of antenatal care initiation (in months)**^**a**^			<0.001*
<eight ANC contacts	4595	5.0±1.5	
Eight or more ANC contacts	1300	3.7±1.4	
**Household headship**			<0.001*
Male	7245 (91.3)	16.5 (15.2–17.8)	
Female	691 (8.7)	27.6 (23.8–31.8)	
**Household wealth quintile**			<0.001*
Poorest	1689 (21.3)	8.1 (6.6–10.0)	
Poorer	1710 (21.6)	10.8 (9.0–13.0)	
Middle	1656 (20.9)	14.9 (12.5–17.8)	
Richer	1524 (19.2)	20.1 (17.2–23.5)	
Richest	1357 (17.1)	37.6 (33.9–41.4)	
**Household size**			<0.001*
1–4	2303 (29.0)	24.4 (21.9–27.1)	
5–8	3562 (44.9)	18.9 (17.2–20.7)	
8+	2071 (26.1)	6.9 (5.6–8.3)	
**Residential status**			<0.001*
Urban	2623 (33.1)	31.2 (28.6–33.9)	
Rural	5313 (66.9)	9.4 (8.3–10.5)	
**Geographical region**			<0.001*
North Central	1365 (17.2)	14.5 (12.1–17.1)	
North East	1692 (21.3)	3.5 (2.6–4.7)	
North West	2497 (31.5)	3.7 (2.8–4.8)	
South East	871 (11.0)	35.6 (31.4–40.1)	
South South	734 (9.2)	36.1 (31.2–41.2)	
South West	777 (9.8)	61.0 (55.6–66.2)	
**Cultural norm for wife beating**			<0.001*
No	5303 (66.8)	22.2 (20.5–24.2)	
Yes	2633 (33.2)	7.7 (6.4–9.1)	
**Community-level poverty**			<0.001*
Low	3922 (49.4)	28.2 (25.6–30.9)	
High	4014 (50.6)	5.9 (4.9–7.0)	
**Community-level media use**			<0.001*
Low	3981 (50.2)	5.7 (4.5–7.1)	
High	3955 (49.8)	29.0 (26.8–31.4)	
**Community-level illiteracy**			<0.001*
Low	3741 (47.1)	32.5 (30.1–34.9)	
High	4195 (52.9)	4.7 (3.8–5.7)	
**Community-level urban residence**			<0.001*
Low	5313 (66.9)	9.4 (8.3–10.5)	
High	2623 (33.1)	31.2 (28.6–33.9)	
**Community-level women’s autonomy**			<0.001*
Low	4050 (51.0)	8.6 (7.3–10.1)	
High	3886 (49.0)	26.8 (24.5–29.3)	

*Significant at p<0.25; a = mean ± standard deviation

### Prevalence of eight or more ANC contacts in Nigeria

**Fig 1:**
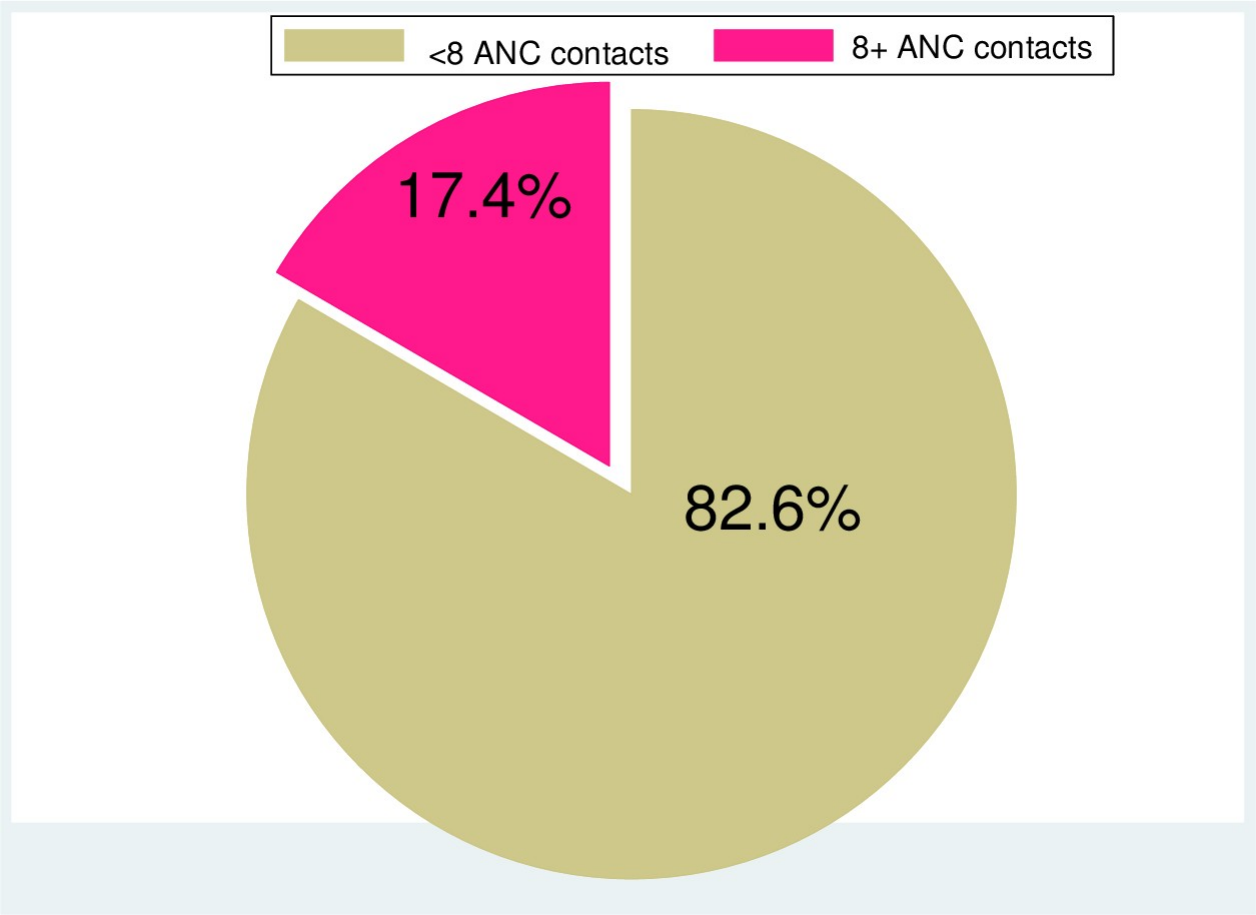
Prevalence of eight or more ANC contacts in Nigeria.

Result from Fig 1 showed the prevalence of eight or more ANC contacts in Nigeria. Approximately 17.4% (95% CI: 16.1%-18.7%) had the optimal ANC contacts as recommended in the new WHO eight ANC guideline.

### Measures of variations (random effects) and model fit statistics

In Table 2, Model V (full model) was selected as the most suitable due to the least AIC and BIC values (1371.30 and 1611.81 respectively). The variations in the odds of eight or more ANC contacts across communities (σ^2^ = 1.32; SE = 0.22) and households (σ^2^ = 0.01; SE = 4.16) were estimated. Results from Median Odds Ratio became the evidence of community contextual factors shaping eight or more ANC contacts. It was estimated that if a women moved to another community or household with a higher probability of eight or more ANC contacts, the median increase in their odds of eight or more ANC contacts would be 3.51 with ICC of 34.5%. MOR equal to unity, indicated no household variance given ICC of 0.0%. In both community and household levels, the explained variances were about 78.3% and 99.8% respectively. This implied that large amount of variances in eight or more ANC contacts have been explained by the household-level factors. Furthermore, VPC for community and household levels were estimated to 18.1% and 72.7% respectively.

**Table 2:** Random effect estimates of individual-, household- and community-level factors associated with eight or more ANC contacts in Nigeria.

Random-effect	Model I	Model II	Model III	Model IV	Model V
**Community-level**					
Variance (SE)	6.09 (1.06)*	1.68 (0.22)*	2.71 (0.54)*	1.43 (0.21)*	1.32 (0.22)
VPC	43.8%	30.2%	39.6%	19.4%	18.1%
Explained variance (PCV)	Reference	72.4%	55.5%	76.5%	78.3%
MOR	330	4.98	13.25	3.91	3.51
ICC	63.0%	46.3%	53.1%	25.6%	34.5%
**Household-level**					
Variance (SE)	4.31 (0.77)*	1.48 x 10^−06^ (1.07)	1.79 (0.68)	1.63 (0.48)	0.01 (4.16)
VPC	29.2%	61.6%	31.3%	41.9%	72.7%
Explained variance (PCV)	Reference	65.7%	58.5%	62.2%	99.8%
MOR	61.28	1.00	5.50	4.71	1.01
ICC	32.0%	0.0%	23.1%	33.1%	0.0%
**Model fit statistics**					
AIC	5900.83	1553.01	5741.17	5077.92	1371.30
BIC	5921.73	1695.13	5810.84	5175.44	1611.81

Model I–empty null model, baseline model without any explanatory variables (unconditional model)

Model II–adjusted for only individual-level factors

Model III–adjusted for only household-level factors

Model IV–adjusted for only community-level factors

Model V–adjusted for individual-, household-, and community-level factors (full model)

VPC Variance Partition Coefficient, AIC Akaike's Information Criterion, BIC Bayesian Information Criterion, PCV Proportional Change in Variance, ICC Intra-class correlation

*Significant at p<0.05

### Measures of associations (fixed effects)

Model V has been selected as the best model because it has the least AIC and BIC values (Table 2). Based on the results, women with at least secondary were 2.46 times as likely to have eight or more ANC contacts when compared with women with no formal education (AOR = 2.46; 95%CI: 1.21, 4.99) (Table 3). Furthermore, women who use media were 2.37 times as likely to have eight or more ANC contacts, when compared with women who do not use media (AOR = 2.37; 95%CI: 1.32, 4.24). In addition, for every unit increase in the time (month) of ANC initiation, there is 53% reduction in the odds of eight or more ANC contacts (AOR = 0.47; 95%CI: 0.40, 0.55). Rural women had 60% reduction in the odds of eight or more ANC contacts, when compared with their urban counterparts (AOR = 0.40; 95%CI: 0.24, 0.66). Geographical region was significantly associated with the odds of eight or more ANC contacts; women from North East and North West had 74% and 79% reduction in the odds of eight or more ANC contacts and women from South East, South South and South West were 2.68, 5.00 and 14.22 times as likely to have eight or more ANC contacts respectively, when compared with women from North Central.

**Table 3:** Fixed effect of individual-, household- and community-level factors associated with eight or more ANC contacts in Nigeria.

Variable	Model I	Model II	Model III	Model IV	Model V
**Individual level factors**					
**Family mobility**					
Internal immigrant		1.00			1.00
Native		1.13 (0.73–1.74)			1.40 (0.91–2.15)
**Religion**					
Christianity		1.00			1.00
Islam		0.62 (0.40–0.96)*			1.26 (0.74–2.14)
ATR/others		2.14 (0.18–24.95)			1.56 (0.14–17.70)
**Number of children ever born**					
1–2		1.00			1.00
3–4		1.01 (0.65–1.56)			1.27 (0.76–2.10)
4+		0.64 (0.36–1.15)			1.27 (0.65–2.46)
**Educational attainment**					
No formal education		1.00			1.00
Primary		3.69 (1.87–7.27)*			1.93 (0.96–3.92)
Secondary+		6.02 (3.18–11.42)*			2.46 (1.21–4.99)*
**Media use**					
No		1.00			1.00
Yes		3.62 (2.15–6.09)*			2.37 (1.32–4.24)*
**Women’s age**					
15–19		1.00			1.00
20–24		1.78 (0.73–4.37)			1.22 (0.49–3.04)
25–29		2.05 (0.83–5.08)			1.00 (0.40–2.51)
30–34		2.47 (0.97–6.34)			0.97 (0.37–2.55)
35–39		2.78 (1.01–7.72)*			1.09 (0.39–3.10)
40–44		5.75 (1.59–20.79)*			2.38 (0.65–8.73)
45–49		3.78 (0.65–22.06)			0.73 (0.11–4.78)
**Wanted child when became pregnant**					
Then		1.00			1.00
Later		1.40 (0.82–2.41)			1.32 (0.76–2.28)
No more		1.64 (0.61–4.37)			1.12 (0.43–2.92)
**Health insurance**					
Not covered		1.00			1.00
Covered		0.74 (0.26–2.14)			0.79 (0.28–2.25)
**Employment status**					
Not working		1.00			1.00
Working		1.68 (1.10–2.58)*			1.31 (0.85–2.03)
**Family type**					
Monogamy		1.00			1.00
Polygyny		1.55 (0.92–2.62)			1.61 (0.91–2.85)
**Intimate partner violence**					
No		1.00			1.00
Yes		0.82 (0.57–1.18)			1.32 (0.90–1.92)
**Women’s autonomy**					
Low		1.00			1.00
Moderate		1.20 (0.74–1.93)			1.04 (0.61–1.78)
High		1.16 (0.73–1.84)			0.78 (0.46–1.33)
**Time of antenatal care initiation (in months)**		0.47 (0.41–0.55)*			0.47 (0.40–0.55)*
**Household-level factors**					
**Household headship**					
Male			1.00		1.00
Female			1.22 (0.83–1.80)		0.87 (0.49–1.54)
**Household wealth quintile**					
Poorest			1.00		1.00
Poorer			1.83 (1.12–2.99)*		0.48 (0.23–0.97)*
Middle			2.60 (1.46–4.65)*		0.57 (0.27–1.18)
Richer			4.34 (2.15–8.78)*		0.61 (0.29–1.29)
Richest			15.03 (4.99–45.26)*		1.19 (0.53–2.65)
**Household size**					
1–4			1.00		1.00
5–8			0.76 (0.57–1.01)		0.88 (0.55–1.40)
8+			0.32 (0.19–0.55)*		1.08 (0.52–2.23)
**Community-level factors**					
**Residential status**					
Urban				1.00	1.00
Rural				0.54 (0.38–0.77)*	0.40 (0.24–0.66)*
**Geographical region**					
North Central				1.00	1.00
North East				0.18 (0.09–0.36)*	0.26 (0.11–0.60)*
North West				0.18 (0.10–0.34)*	0.21 (0.09–0.50)*
South East				3.39 (1.93–5.94)	2.68 (1.37–5.23)*
South South				2.75 (1.61–4.69)*	5.00 (2.40–10.40)*
South West				18.77 (7.46–47.23)*	14.22 (7.12–28.41)*
**Cultural norm for wife beating**					
No				1.00	1.00
Yes				0.80 (0.60–1.06)	0.76 (0.47–1.22)
**Community-level poverty**					
Low				1.00	1.00
High				0.55 (0.37–0.82)*	0.94 (0.52–1.73)
**Community-level media use**					
Low				1.00	1.00
High				1.30 (0.88–1.92)	0.60 (0.32–1.13)
**Community-level illiteracy**					
Low				1.00	1.00
High				0.48 (0.31–0.73)*	0.99 (0.54–1.82)
**Community-level women’s autonomy**					
Low				1.00	1.00
High				1.13 (0.82–1.55)	0.96 (0.57–1.63)

Model I–empty null model, baseline model without any explanatory variables (unconditional model)

Model II–adjusted for only individual-level factors

Model III–adjusted for only household-level factors

Model IV–adjusted for only community-level factors

Model V–adjusted for individual-, household-, and community-level factors (full model)

*Significant at p<0.05

## Discussion

Three years have passed since the endorsement of the minimum eight ANC contacts guideline. In Nigeria, this study stands in the frontline to determine eight or more ANC coverage and the factors that influence ANC contacts among women. Specifically, the guideline stipulates that the first ANC contact takes place within the first trimester of the pregnancy, that is, 12 weeks of gestation, while the next two contacts take place during the second trimester of the pregnancy that is between the 20^th^ and 26^th^ gestation weeks. About five contacts are due to take place during the third trimester, that is between 30^th^ through the 40^th^ week of gestation [35].

Our study revealed that 17.4% of women had the updated WHO recommendation of eight ANC contacts. This shows that compliance with the updated recommendation is low, probably because it may not have been adopted as a national policy. This study reported a higher prevalence than a previous study conducted using Benin population-based data which also examined the coverage of eight or more ANC visits [36]. Similarly, the observed prevalence is slightly higher than results from a previous multi-country study that revealed 15.3% of mothers had eight or more antenatal contacts; with the first contact in the first trimester of pregnancy. Unfortunately, that study utilized data collected before the endorsement of the eight ANC guideline [37]. Therefore, the basis for the eight or more ANC in that study is not certain. In another study, only 6.1% of mothers reported eight or more ANC contacts [38]. Also, the data used was collected before the endorsement of the WHO eight ANC guideline and the findings cannot be credited to the implementation or non-implementation of the guideline. Since the WHO recommendations were released in 2016 [8], it could be that before 2016, women who had eight or more ANC contacts were probably high-risk pregnancies that required close monitoring by a healthcare provider. With the new guideline [8,35], healthcare providers will have the basis to schedule a woman with normal pregnancy for eight or more contacts.

Among the individual-level factors examined, educational attainment, media use, and timing to ANC initiation were significantly associated with eight or more ANC contacts in Nigeria. The place of residence and geographical region were also significant factors at the community level. We found that education had a positive association with having eight or more ANC contacts. Women who had primary and secondary or higher education had increased odds of eight or more ANC contacts compared with women who had no formal education. Similarly, a study in Bangladesh found education to be positively associated with eight or more ANC contacts during pregnancy [38]. Studies that have examined 4 or more ANC contacts in different settings, have consistently reported that women with higher levels of education have a higher prevalence and greater odds of 4 or more ANC contacts [39–47]. Maternal education is associated with the use of basic maternal health services including ANC [40,48] and lack of formal education has been associated with under-utilization or non-use of ANC [12,49]. Maternal education can improve health care literacy; thus, they are more aware of danger signs and may be able to identify signs of pregnancy complications. Also educated women may be more appreciative of the advantages of utilizing health care services including ANC [41,50–53]. Furthermore, it can enhance women’s socioeconomic opportunities and status [52] decision-making power [41,52,54,55], and the confidence to take actions about their health [25,43,56].

In addition, media use and early timing to ANC initiation were significantly associated with eight or more ANC contacts. Previous studies have reported similar findings with regards to eight or more ANC contacts [38] and 4 or more ANC contacts [40,41,47,57,58]. Media access may improve women’s enlightenment positively and ANC utilization via health education and influences maternal health care seeking behaviour [41,59,60]; pregnant women who listen to the radio, watch the television or read the newspaper, are more likely to have important health information and messages about maternal health services and advantages of utilizing them [38,41,44,60]. This underscores the importance of social and behavioural change communication to increase awareness and create demand for the use of maternal health services [12]. Similarly, the findings from previous studies showed that women enlightenment and early ANC initiation were significantly associated with eight or more ANC contacts [36,61]. There is no doubt, that early booking for ANC would result in optimal number of ANC contacts during pregnancy as those who start early will have higher possibility of achieving optimal ANC visits.

At the community level, we found that having eight or more ANC contacts differed significantly between geographical regions of the country, with the highest proportions reported in the South West and the lowest was observed in the North East. Although there is paucity of studies that have examined eight or more ANC visits in Nigeria, previous studies that explored 4 or more ANC visits found a similar pattern of geographic variation, with higher levels of ANC use in the South West and South East, and lower levels of use in the Northern regions [11,16,39,62]. In their study, Adewuyi *et al*. found that the highest proportion of mothers that had 4 or more ANC visits were in Southwest of Nigeria; only 10.2% reported having less than 4 or more ANC contacts [12]. Differences in socioeconomic development, educational attainment, accessibility to health facilities may be responsible for the observed geographical variations [39,63]. Also, security challenges in parts of the North (insurgency) and South-South (militancy) may contribute to the observed variation [12,64,65].

Women residing in rural areas had reduction in eight or more ANC contacts compared with their urban counterparts. In a previous study, Islam *et al* reported similar finding [38]. Other studies of determinants of 4 or more ANC contacts have reported similar findings [12,41,43,45,47,66]. There are significant differentials in rural-urban utilization of maternal health services including ANC due to inequities in the distribution of accessible health resources between rural and urban areas [11,12,41,48,67]. Where facilities exist, they may be inaccessible due to poor road network, inefficient transport system or very far distances [12]. Furthermore, rural areas are inadequately financed, and it is difficult to attract and retain health workers in such places [11]. Also, women residing in rural areas may have lower socioeconomic status than their counterparts in urban areas, which might make utilization of ANC services less likely [45,68]. Women in rural areas may be more influenced by cultural beliefs and social norms that discourage utilization of skilled maternal care services [56].

This study provides empirical data on the coverage of the WHO recommended eight or more ANC contacts during pregnancy. This information can inform policy makers in drawing up national guidelines on ANC utilization, which are in line with WHO recommendations. However, designing the guidelines may not be enough to ensure adherence. Thus, there is need to ensure those guidelines reach the end-users, from the pre-service trainees to the in-service health workers. This study also highlights the gap in attainment of the recommended eight or more ANC contacts with regards to area of residence. Thus, ANC services should be provided to women residing in the hard-to-reach areas through establishment and strengthening of primary health care (PHC). Also making ANC services subsidized or free, for example through the basic health care provision fund, can serve as an incentive to encourage ANC attendance. Also important is the need for strategically designed health promotion programs that utilize locally contextualized social behaviour change communication (SBCC) messages to create awareness and increase demand for ANC services. As the present study explored the subject from the client’s perspective, future research can examine the demand-side factors of ANC contacts among women and/or ANC coverage from the health workers perspective, both quantitatively and qualitatively.

## Strengths and limitations

The major strength of this study is that it has become the foremost population-based study on eight or more ANC contacts in Nigeria, therefore, it will serve as a stimulus and benchmark for further studies. In addition, the use of nationally representative data makes the findings generalizable to women of reproductive age in Nigeria. The use of data collected after the endorsement of the new eight or more ANC contacts, make this study of plausible conclusion. Conversely, the use of cross-sectional data established that only associations and no causal relationships are reported. In addition, we could not measure the sources of demand-side unobserved heterogeneity across the secondary data and this could cause some bias in our findings. The unavailability of relevant variables such as women’s perception and awareness of skilled maternal health care was a major limitation in the DHS data. Hence, we considered supply-side limitations of data to those issues related to health care delivery. More so, recall bias could have occurred in this study due to self-reported number of ANC contacts, except when ANC contacts were determined using hospital cards.

## Conclusion

In this study, we have revealed that achievement of the WHO recommended eight or more ANC contacts was poor and can be influenced multilevel factors. Therefore, there is need for a national adoption of the WHO recommendation and to encourage implementation. There should be concerted efforts from health care stakeholders in the improvement of maternal socioeconomic status, as well as create awareness among the populace for optimal utilization of ANC. Interventions to improve ANC attendance should also target barriers linked with inequities and inequalities in ANC utilization as related to geographical residence.
